# Integrated RPA–CRISPR/Cas12a Technology for Rapid Detection of *Salmonella enterica*

**DOI:** 10.3390/diagnostics16091371

**Published:** 2026-04-30

**Authors:** Ainur Akimbekova, Aisha Shaizadinova, Meruyert Amanzholova, Aitbay Bulashev, Sailau Abeldenov

**Affiliations:** 1 Institute of Animal Science and Veterinary, Saken Seifullin Kazakh Agrotechnical University, Astana 010000, Kazakhstan; aynurakimbekova12@gmail.com (A.A.);; 2 National Center for Biotechnology, Astana 010000, Kazakhstan; 3 Faculty of Biology and Biotechnology, Al-Farabi Kazakh National University, Almaty 050040, Kazakhstan

**Keywords:** *Salmonella enterica*, portable fluorescence visualization, RPA (Recombinase Polymerase Amplification), CRISPR/Cas12a, isothermal detection, virulence genes

## Abstract

**Background/Objectives:** Rapid identification of foodborne pathogens is of high practical significance because it enables prompt epidemiological response, timely patient management, and effective sanitary control of food products. In this study, we developed an integrated molecular platform combining recombinase polymerase amplification (RPA) with CRISPR/Cas12a technology for rapid, sensitive, and specific detection of *Salmonella enterica*. **Methods:** Four virulence genes (*sirA*, *stn*, *siiD*, and *pagN*) were selected as targets to ensure reliable pathogen identification. Reaction conditions were optimized using the *Moraxella bovoculi* Cas12a (MbCas12a) nuclease. The study focused on integrating isothermal amplification with a custom-engineered hardware solution for visual fluorescence detection. **Results:** The developed method demonstrated sensitive and specific detection, with no cross-reactivity to non-target microorganisms. Optimization allowed for a substantially reduced assay time of approximately 30 min. As a result, a portable fluorescence visualization approach was developed, featuring a 3D-printed housing and an integrated ultraviolet light source for direct visual fluorescence detection. This allows rapid differentiation of samples without specialized laboratory equipment, making it suitable for field applications. **Conclusions:** The combination of isothermal amplification, MbCas12a-based detection, and the portable fluorescence visualization approach provides a versatile platform for rapid diagnostics and food safety monitoring. This approach has strong potential to improve public health outcomes and enhance the resilience of food supply chains by enabling accessible, field-deployable pathogen detection.

## 1. Introduction

In recent years, food safety has emerged as one of the most critical global challenges, as the increasing number of disease outbreaks and the chronic exposure to foodborne pathogens continue to threaten public health and disrupt global supply chains [1]. Among these pathogens, *Salmonella enterica* is recognized as one of the most hazardous due to its widespread presence across diverse food matrices and its ability to cause salmonellosis a disease ranging from self-limiting gastroenteritis to severe systemic infections with substantial morbidity and mortality [2,3]. Given the growing economic and healthcare burden associated with salmonellosis, there is an urgent need to develop rapid, reliable, and cost-effective molecular diagnostic technologies capable of detecting even minimal levels of contamination in food products [4]. This also aligns with recent research emphasizing the importance of sensitive diagnostic tools for *Salmonella enterica* circulating in Kazakhstan. Previous studies reported successful expression of recombinant *S. enterica* OmpX protein for serological detection [5] and provided genome-level insights into *Salmonella Abortusequi* strains associated with equine abortions [6].

Traditional culture-based methods, the gold standard for pathogen identification, require prolonged incubation periods and involve labor-intensive procedures, making them impractical for real-time monitoring in the rapidly evolving environment of food production [7]. Although the introduction of polymerase chain reaction (PCR) has substantially accelerated diagnostics and improved sensitivity, this method still presents several limitations—it requires expensive instrumentation and strict temperature control. Furthermore, these traditional approaches often rely on complex laboratory infrastructure and trained personnel, reducing their accessibility in decentralized or resource-limited settings [8]. In light of these challenges, considerable research attention has shifted toward next-generation molecular detection strategies that combine isothermal amplification with CRISPR-based diagnostics, offering significant improvements in speed, sensitivity, and operational simplicity [9,10,11,12].

Isothermal amplification methods such as loop-mediated isothermal amplification (LAMP) and recombinase polymerase amplification (RPA) have emerged as promising alternatives to conventional PCR owing to their ability to amplify target DNA at a constant temperature [13,14]. These methods significantly reduce analysis time and eliminate the need for thermocyclers, thus enabling rapid, field-deployable diagnostics. Moreover, the intrinsic simplicity of isothermal amplification and its minimal equipment requirements make these assays particularly suitable for decentralized environments where immediate pathogen detection is essential for preventing the spread of foodborne contaminants. Recent studies have demonstrated that these approaches can achieve limits of detection as low as a few colony-forming units (CFU) per milliliter, underscoring their potential as effective early-warning tools in food safety management [15,16,17].

Building on the advances of isothermal amplification, the integration of CRISPR/Cas-based detection represents a significant technological breakthrough in molecular diagnostics [18,19]. Among the various CRISPR systems available, CRISPR/Cas12a has garnered considerable attention due to its dual functionality: target recognition and collateral cleavage of single-stranded DNA reporters upon activation [20,21].

Recent studies have successfully applied Cas12a for *Salmonella* detection, reporting high sensitivity and specificity [22,23,24,25,26,27,28,29,30,31,32,33]. However, the study of existing literature reveals that most current RPA-CRISPR assays for *Salmonella* rely on the widely used *Lachnospiraceae bacterium* Cas12a (LbCas12a) and target only a single genetic locus (e.g., *invA* or *ssrv*) [34]. While effective, reliance on a single target may limit diagnostic flexibility in cases of genomic variation [35]. Furthermore, many existing platforms require 60 to 90 min for a full diagnostic cycle.

To expand the diagnostic toolkit for foodborne pathogens, this study utilizes the Cas12a nuclease derived from *Moraxella bovoculi* (MbCas12a). The implementation of MbCas12a allowed for the establishment of a streamlined detection framework, which we systematically evaluated and optimized for four distinct virulence targets: *stn*, *siiD*, *sirA*, and *pagN*. The optimized protocol achieves pathogen identification within 30 min, offering a competitive turnaround time compared to previously reported CRISPR-based assays. When integrated with a portable fluorescence visualization approach, this MbCas12a-based multiple target strategy provides a robust and adaptable solution for decentralized food safety monitoring [36].

The aim of this study was to develop and validate RPA-CRISPR/Cas12a-based detection system, evaluate its analytical sensitivity and specificity for the selected targets, and determine the optimal conditions for reliable molecular diagnostics.

## 2. Materials and Methods

### 2.1. Bacterial Strains, Isolates and Genetic Targets

The *Salmonella enterica* subsp. *enterica* serovar *Typhimurium* strain ATCC 13311 was used as the primary reference strain. To further validate the assay under realistic conditions, four bacterial isolates were obtained from poultry farms and provided by the Laboratory of Applied Genetics. These included two confirmed strains of *S. enterica* and two *Salmonella* spp. isolates. Reference nucleotide sequences for the target genes *stn*, *siiD*, *sirA*, and *pagN* were obtained from the NCBI GenBank database. These genes, associated with pathogenicity and virulence, were selected as diagnostic markers for *S. enterica* identification. *E. coli DH5α* strains were employed for gene transformation via the heat-shock method during plasmid template preparation. The analytical specificity of the assay was assessed using genomic DNA extracted from the following non-target bacterial strains: *Acinetobacter baumannii*, *Pseudomonas aeruginosa*, *Escherichia coli*, *Staphylococcus aureus*, *Streptococcus agalactiae*, and *Klebsiella pneumoniae*. All strains and isolates were sourced from the laboratory culture collection.

### 2.2. Reagents

All reagents used in this study were purchased from Sigma-Aldrich (St. Louis, MO, USA), AppliChem (Darmstadt, Germany), Promega (Madison, WI, USA), and Amresco (Solon, OH, USA) and were of «molecular biology grade». The MbCas12a protein was expressed and purified in-house using standard affinity and size-exclusion chromatography protocols. Protein purity and concentration were assessed by SDS-PAGE and spectrophotometric analysis. Fluorescent reporter probes FAM-TTATT-BHQ were synthesized by Lumiprobe RUS LLC (Moscow, Russia) and the National Center for Biotechnology (Astana, Kazakhstan). Reporter molecules contained a 5′ FAM fluorophore and a 3′ BHQ quencher. All reactions were performed in NEBuffer™ 2.1 (10×) (New England Biolabs, Ipswich, MA, USA).

### 2.3. DNA Extraction and Sample Preparation

For the reference strain *S. enterica* ATCC 13311 and non-target bacterial strains, genomic DNA was isolated using the GeneJET Genomic DNA Purification Kit (Thermo Scientific, Waltham, MA, USA) according to the manufacturer’s instructions. The GeneJET protocol included: cell lysis in a detergent- and proteinase K-containing buffer, incubation to disrupt cell membranes and denature proteins, addition of a precipitation buffer, transfer of the lysate onto a spin column, DNA binding, washing steps, and final elution in nuclease-free water. Purified genomic DNA samples were stored at −20 °C until further analysis. For bacterial isolates, a simplified thermal lysis method was applied. Bacterial cells were resuspended in saline solution, heated at 95 °C for 5 min, and subsequently centrifuged at 10,000× *g* for 5 min. The resulting supernatant, containing released genomic DNA, was used directly as a template for the RPA–CRISPR/Cas12a assays.

### 2.4. Oligonucleotides

Synthetic oligonucleotides used in the experimental procedures were synthesized at the National Center for Biotechnology (Astana, Kazakhstan) (Table 1).

### 2.5. Construction of Positive Control Plasmids

To generate plasmid constructs used as positive controls, the *sirA* (191 bp), *stn* (154 bp), *siiD* (826 bp) and *pagN* (440 bp) genes were amplified using Phusion DNA polymerase by polymerase chain reaction (PCR). The resulting PCR products were verified for correct amplification and subsequently cloned using the CloneJET PCR Cloning Kit (Thermo Scientific). The obtained plasmids were sequenced to confirm the absence of mutations. These plasmids containing positive controls were used for optimization of isothermal RPA reactions and for determining assay sensitivity.

### 2.6. Primer Design

Design of primers specific to the *stn*, *siiD*, *sirA* and *pagN* genes of *Salmonella* spp. was performed using reference sequences from NCBI GenBank. Based on these sequences, primers were designed using Vector NTI v11 and Benchling (Benchling Inc., San Francisco, CA, USA; https://www.benchling.com, accessed 3 March 2025) software tools, ensuring appropriate melting temperature (Tm), GC content, and absence of primer-dimer formation. Primers of 24–30 nucleotides in length with Tm values of 60–65 °C and predicted amplicon sizes of 100–1000 bp were selected for further cloning and sequencing.

### 2.7. crRNA Design and Synthesis

To identify suitable genomic targets in *Salmonella* spp., loci containing the required Cas12a PAM motif (TTTN) were screened. Based on these sequences, crRNAs targeting the *stn*, *siiD*, *sirA* and *pagN* genes were designed. Each crRNA contained a structural “handle” for Cas12a interaction and a target-specific spacer region complementary to the corresponding genomic site. crRNA synthesis was performed using the HiScribe T7 Quick High Yield RNA Synthesis Kit (New England Biolabs, Ipswich, MA, USA). Double-stranded DNA templates assembled from synthetic oligonucleotides and containing the T7 promoter and crRNA target sequence served as transcription templates. *In vitro* transcription was carried out according to the manufacturer’s instructions. Following synthesis, transcription mixtures were treated with DNase I to remove DNA templates. Purification of crRNAs was performed using the Monarch RNA Cleanup Kit (New England Biolabs). This ensured efficient removal of enzymes, nucleotides, and DNA contaminants while preserving RNA integrity and stability. The quality of purified crRNAs was evaluated spectrophotometrically using A260/A280 ratios. Only high-purity, non-degraded RNA samples were used for subsequent assembly of Cas12a-crRNA ribonucleoprotein complexes.

### 2.8. RPA Reaction Conditions

Amplification of the *stn*, *siiD*, *sirA* and *pagN* genes was performed using the TwistAmp Basic Kit (TwistDx, Cambridge, UK). For each reaction, 2.4 μL of 10 μM forward and reverse primers, 29.5 μL of rehydration buffer, and 13.2 μL of template solution with water were combined, bringing the total volume to 47.5 μL. The lyophilized RPA enzyme pellet was activated by adding 2.5 μL of 280 mM magnesium acetate bringing the final reaction volume to 50 μL. Amplification was carried out at 37 °C for 20 min according to the manufacturer’s protocol.

### 2.9. Recombinant Cas12a Protein Used in Fluorescent Assay

In this study, Cas12a derived from *Moraxella bovoculi* was used, which has been successfully validated in our previous research. This enzyme demonstrated high efficiency and reliability in the detection of various pathogens, including *Escherichia coli* in bovine mastitis milk samples [37], *Staphylococcus aureus* in cultured milk samples [38], and *Alternaria* spp. in wheat grain samples [39]. Proteins were expressed in *E. coli* ArcticExpress (DE3) using pET-28c-based plasmid constructs and induced with IPTG at 20–25 °C. Soluble Cas12a was extracted from the cell lysate and purified via a two-step procedure comprising immobilized metal affinity chromatography (IMAC) followed by heparin affinity chromatography. Protein purity and identity were confirmed by SDS-PAGE and LC-MS/MS. Purified proteins were stored at −20 °C in 50% glycerol until use in diagnostic assays.

### 2.10. CRISPR/Cas12a-RPA Fluorescent Assay

To assemble MbCas12a-crRNA complexes, 100 nM MbCas12a (laboratory collection) was mixed with 100 nM crRNA and incubated at 25 °C for 15 min. Cas12a trans-cleavage reactions were performed in a total volume of 30 μL. The reaction mixture contained NEBuffer 2.1 (New England Biolabs), 100 nM crRNA, 100 nM MbCas12a (origin: *Moraxella bovoculi*, laboratory collection), 8 μM fluorescent reporter, and 3 μL of RPA-amplified product. Incubation was carried out at 37 °C for 10 min. Fluorescent output was visualized using a Vilber Lourmat transilluminator (Paris, France) at a wavelength of 320 nm. Fluorescence images were captured using a smartphone camera and stored for downstream analysis.

### 2.11. Determination of Analytical Sensitivity

To determine the limit of detection, RPA reactions were performed using a series of tenfold dilutions of positive control plasmid DNA containing the target gene fragment. DNA concentrations ranged from 10^10^ to 10^0^ copies per reaction for plasmid DNA. The number of DNA copies was calculated based on the initial plasmid concentration and molecular weight. The LoD was defined as the lowest concentration of target DNA that produced a detectable fluorescence signal under the tested experimental conditions, based on serial dilution analysis. This experimental approach enabled identification of the minimum DNA copy number at which the assay retained reliable detection capability.

### 2.12. Determination of Analytical Specificity

Analytical specificity of the RPA assay was evaluated experimentally using *Salmonella enterica* genomic DNA as the positive control. Reactions were performed using the TwistAmp Basic Kit (TwistDx, Cambridge, UK). Specificity testing included DNA samples from other bacterial species described in Section 2.1.

### 2.13. Real-Time Fluorescence Monitoring

Real-time monitoring of fluorescence was performed using the CFX96 Real-Time PCR System (Bio-Rad, Hercules, CA, USA). Fluorescence was recorded in the FAM channel as relative fluorescence units (RFU) at 60-s intervals for 30 min.

### 2.14. Statistical Analysis

Statistical analysis was applied to experiments involving real samples, where results are presented as mean values ± standard deviation (SD). Data were analyzed using GraphPad Prism v8 software (GraphPad Software, San Diego, CA, USA).

## 3. Results

### 3.1. Amplification of Target Sequences by RPA

For the rapid identification of *Salmonella enterica*, isothermal amplification by RPA was performed, a method characterized by high sensitivity and rapid reaction under constant temperature conditions. The reaction was conducted at 37 °C for 20 min, ensuring efficient and robust amplification of the target DNA regions.

Electrophoretic analysis of the amplification products in a 2% agarose gel revealed distinct bands corresponding to the expected fragment sizes for the *sirA* (191 bp), *stn* (154 bp), *siiD* (826 bp), and *pagN* (440 bp) genes. During the determination of the limit of detection, electrophoresis confirmed the formation of amplification products with lengths strictly matching the expected sizes for each gene examined, indicating high specificity and efficiency of the RPA reaction.

**Figure 1:**
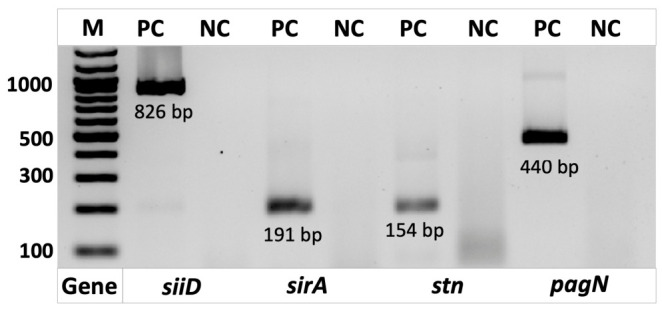
Electrophoretic analysis of RPA products. The electropherogram shows the amplification products of the target genes *siiD* (826 bp), *sirA* (191 bp), *stn* (154 bp) and *pagN* (440 bp). Samples marked as «PC» correspond to positive control reactions containing *Salmonella enterica* DNA, whereas samples marked as «NC» represent negative controls (no template DNA). The positive samples display amplification products of the expected sizes, confirming the proper primer design and the specificity of the RPA amplification.

To verify the efficiency and specificity of the designed primers, RPA analysis was performed using *Salmonella enterica* DNA as a positive control. Electrophoretic analysis of the amplification products (Figure 1) showed clear bands of the expected sizes for the target pathogen genes used in this study. No amplification products were observed in the negative control reactions, indicating the absence of nonspecific primer binding and confirming the high specificity of the RPA reaction.

### 3.2. Assessment of Trans-Cleavage Activity of Target Sequences

The trans-cleavage activity of the developed RPA-CRISPR/Cas12a system was evaluated using plasmid DNA of *Salmonella enterica* as a positive control, while nuclease-free water served as a negative control (NC). Following isothermal amplification (RPA), reaction products were incubated with the pre-assembled Cas12a/crRNA complex specific to each target gene (*stn*, *siiD*, *sirA* and *pagN*). Upon completion of the incubation, reaction outcomes were recorded using a Vilber Lourmat transilluminator. The presence of intense fluorescence indicated successful Cas12a activation and specific recognition of the target DNA. To confirm the effectiveness of the developed detection system, a combined RPA-CRISPR/Cas12a assay was performed for the four virulence genes investigated in this study. Following isothermal amplification (RPA), the reaction products were incubated with the Cas12a/crRNA complex targeting the corresponding gene regions.

To identify the most efficient PAM motif for Cas12a-mediated detection, all TTTN PAM candidates located within each target amplicon were screened experimentally using the RPA-CRISPR/Cas12a assay. The following PAM sequences were evaluated for the four virulence genes: *sirA*—TTTC, TTTG; *stn*—TTTC, TTTT; *siiD*—TTTA, TTTT; and *pagN*—TTTA, TTTT, TTTC, TTTG. Each candidate PAM site was tested under identical reaction conditions, and the resulting fluorescence intensities were compared to determine Cas12a activation efficiency.

**Figure 2:**
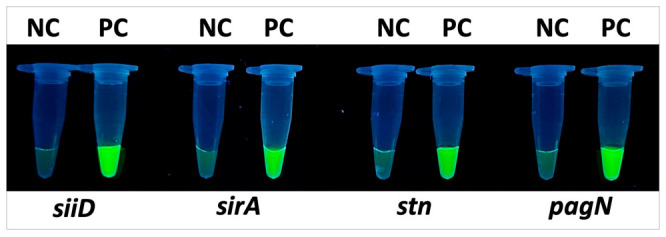
Fluorescent visualization of the RPA-CRISPR/Cas12a assay results for detection of the *stn*, *siiD*, *sirA* and *pagN* genes. Negative control reactions are indicated as NC, and positive controls as PC. Bright green fluorescence is observed in the positive samples, resulting from activation of the Cas12a complex and cleavage of the FAM fluorescent reporter, confirming the presence of the target sequence. No fluorescence is detected in the negative controls.

Based on this analysis, the most productive PAM motifs were selected for subsequent assay development: *sirA*—TTTG, *stn*—TTTC, *siiD* and *pagN*—TTTA. These PAM sequences consistently yielded the highest fluorescence output and were therefore used in all downstream analytical sensitivity and specificity experiments. The results were visualized under UV illumination based on FAM fluorescence. As shown in Figure 2, all positive reactions («PC») exhibited distinct green fluorescence, indicating successful Cas12a activation and cleavage of the fluorescent reporter. No fluorescence was observed in the negative samples («NC»), demonstrating the high specificity of the system and the absence of cross-reactivity.

These results confirm that the developed RPA-CRISPR/Cas12a system effectively detects the virulence genes *stn, siiD, sirA* and *pagN*, providing a rapid, reliable, and visually interpretable assay suitable for molecular diagnostics.

### 3.3. Assessment of the Analytical Sensitivity of TARGET Sequences

The analytical sensitivity of the developed RPA-CRISPR/Cas12a system was evaluated for four virulence genes (*sirA*, *stn*, *siiD* and *pagN*) of *Salmonella enterica*. To evaluate the analytical sensitivity of the target genes a series of experiments was conducted using serial dilutions of a positive control sample (plasmid DNA containing the corresponding fragments). The initial concentrations were as follows: plasmid DNA—*sirA*: 32 ng/µL, *stn*: 27.6 ng/µL, *siiD*: 57.9 ng/µL, *pagN*: 61.5 ng/µL. Dilutions were prepared in the range of 10^10^ to 0 copies per reaction for plasmid DNA.

RPA amplification was carried out using the standard TwistAmp Basic Kit protocol, and reaction products were incubated with the CRISPR/Cas12a complex in the presence of a FAM-labeled reporter for 10 min at 37 °C. Detection was assessed by three complementary methods: electrophoresis of RPA products, visual fluorescence observation, and real-time fluorescence monitoring.

#### 3.3.1. *siiD* Gene

**Figure 3:**
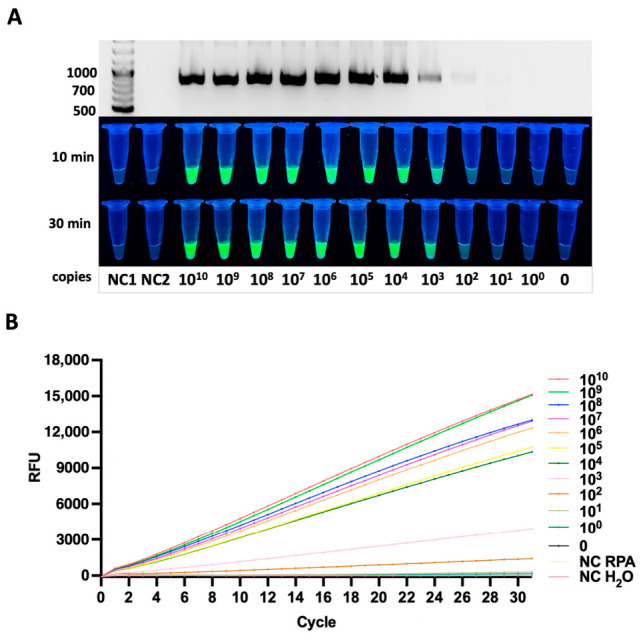
Analytical sensitivity of the RPA-CRISPR/Cas12a assay for detection of the *siiD* gene. (**A**) Detection of serial dilutions of plasmid DNA, illustrated by RPA gel electrophoresis (**upper panel**) and fluorescence readout at 10 and 30 min (**lower panels**). The progressive increase in fluorescence intensity corresponds to decreasing template DNA quantities, indicating the sensitivity threshold of the assay for the plasmid sample. (**B**) Real-time fluorescence analysis for plasmid DNA, showing concentration-dependent signal increase and no amplification in negative controls.

The *siiD* gene was detected with a limit of 10^2^ copies per reaction. Clear amplification products were observed, and strong fluorescence signals were recorded within 10–30 min of the Cas12a reaction. The *siiD* target contained the TTTA PAM sequence, and the fluorescence signal remained clearly distinguishable at the lowest detectable concentration (Figure 3A).

The kinetics of fluorescence development showed that samples with higher copy numbers generated rapid signal accumulation, whereas lower concentrations produced a slower but still detectable fluorescent increase within the 30-min incubation period. Real-time fluorescence monitoring revealed concentration-dependent signal accumulation, allowing clear discrimination between positive samples and negative controls within 30 min. The fluorescence curves demonstrated a clear gradient of signal intensities corresponding to template concentration, confirming a strong quantitative relationship between DNA copy number and Cas12a-mediated reporter cleavage (Figure 3B). Importantly, all no-template controls (NTCs) remained flat throughout the entire measurement period, indicating excellent assay specificity and the absence of nonspecific amplification or background Cas12a activation.

#### 3.3.2. *pagN* Gene

**Figure 4:**
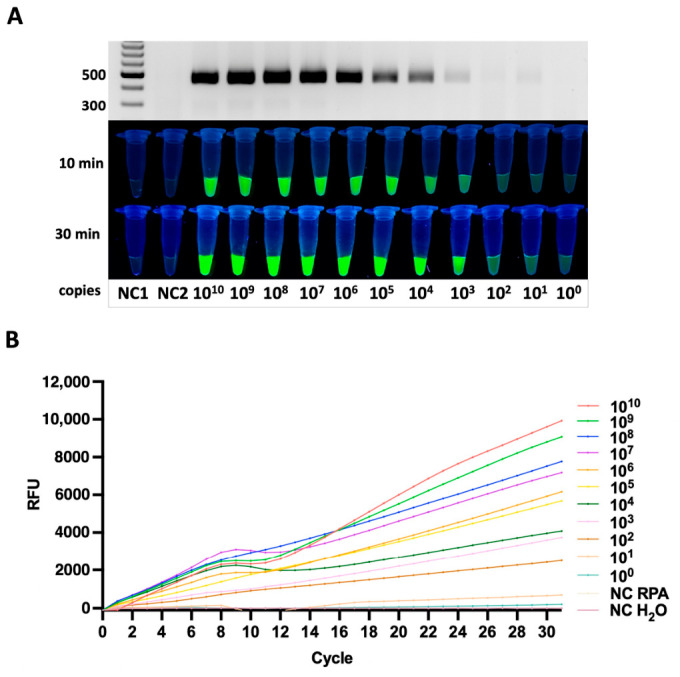
Analytical sensitivity of the RPA-CRISPR/Cas12a assay for detection of the *pagN* gene. (**A**) Detection of serial dilutions of plasmid DNA, illustrated by RPA gel electrophoresis (**upper panel**) and fluorescence readout at 10 and 30 min (**lower panels**). The progressive increase in fluorescence intensity corresponds to decreasing template DNA quantities, indicating the sensitivity threshold of the assay for the plasmid sample. (**B**) Real-time fluorescence analysis for plasmid DNA, showing concentration-dependent signal increase and no amplification in negative controls.

For the *pagN* gene, the assay also reached a detection limit of 10^2^ copies per reaction. A rapid and intense fluorescence signal was detected as early as 10 min after initiating the Cas12a reaction, and real-time monitoring demonstrated a concentration-dependent increase in signal within 30 min. The *pagN* target contained the TTTA PAM sequence (Figure 4A,B).

The fluorescence intensity for *pagN* was nearly comparable to that of *siiD*, suggesting similar efficiency in RNP complex formation and Cas12a activation for these two targets.

#### 3.3.3. *sirA* Gene

**Figure 5:**
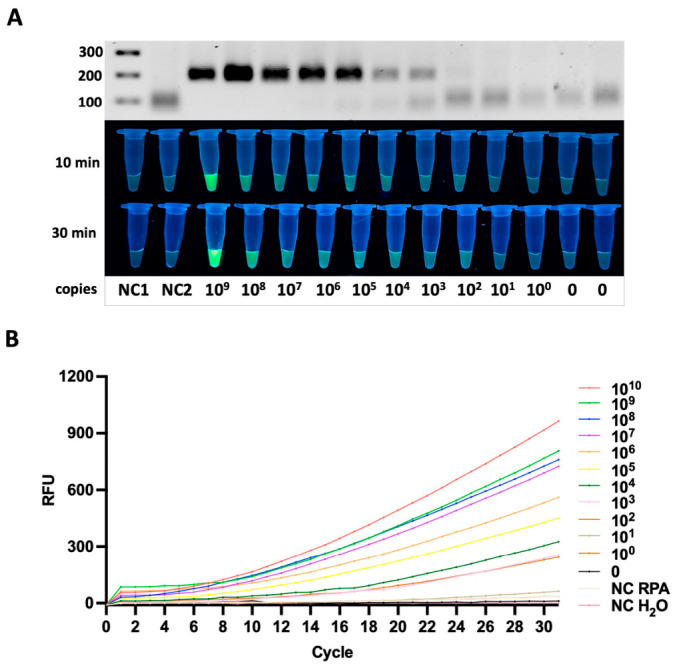
Analytical sensitivity of the RPA-CRISPR/Cas12a assay for detection of the *sirA* gene. (**A**) Detection of serial dilutions of plasmid DNA, illustrated by RPA gel electrophoresis (**upper panel**) and fluorescence readout at 10 and 30 min (**lower panels**). The progressive increase in fluorescence intensity corresponds to decreasing template DNA quantities, indicating the sensitivity threshold of the assay for the plasmid sample. (**B**) Real-time fluorescence analysis for plasmid DNA, showing concentration-dependent signal increase and no amplification in negative controls.

Similarly, the *sirA* gene was reliably detected down to 10^2^ copies per reaction. Although stable RPA amplification was achieved, the fluorescence intensity was moderate compared to *siiD* and *pagN*. The *sirA* target carried the TTTG PAM sequence, and a consistent fluorescence signal was observed across the tested concentrations (Figure 5A).

Nevertheless, all *sirA*-positive samples demonstrated fluorescence values that were reproducibly higher than the baseline signal of the negative controls throughout the monitoring period (Figure 5B). The separation between positive and negative reactions remained stable, confirming the specificity of Cas12a activation for this target.

Despite generating comparatively lower fluorescence output than the *siiD* and *pagN* assays, the *sirA*-directed RPA-CRISPR/Cas12a system demonstrated dependable performance. The observed sensitivity and specificity indicate that *sirA* represents a diagnostically valid marker for species-specific detection of *Salmonella enterica* using the developed assay platform.

#### 3.3.4. *stn* Gene

**Figure 6:**
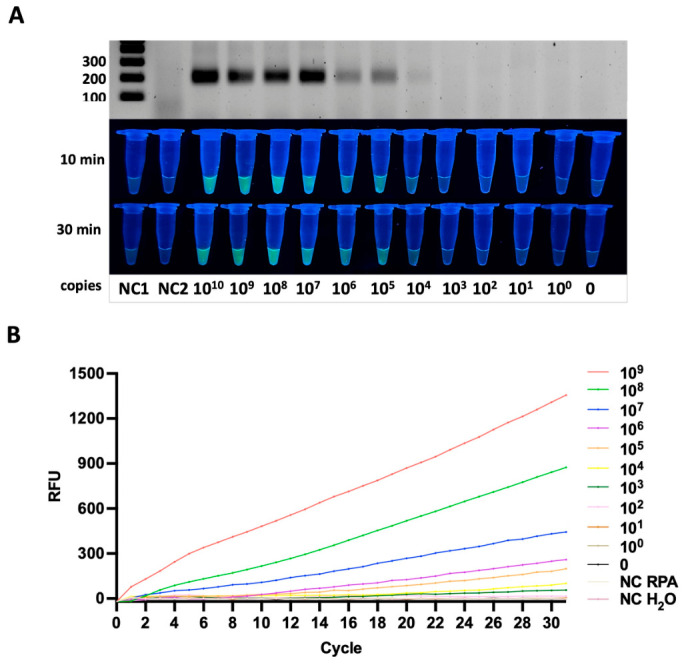
Analytical sensitivity of the RPA-CRISPR/Cas12a assay for detection of the *stn* gene. (**A**) Detection of serial dilutions of plasmid DNA, illustrated by RPA gel electrophoresis (**upper panel**) and fluorescence readout at 10 and 30 min (**lower panels**). The progressive increase in fluorescence intensity corresponds to decreasing template DNA quantities, indicating the sensitivity threshold of the assay for the plasmid sample. (**B**) Real-time fluorescence analysis for plasmid DNA, showing concentration-dependent signal increase and no amplification in negative controls.

In contrast, the *stn* gene exhibited a lower analytical sensitivity, with a detection limit of 10^4^ copies per reaction. At this concentration, the fluorescence signal was weaker but remained distinguishable from the background. The *stn* target contained the TTTC PAM sequence, and amplification was consistently confirmed by both electrophoretic analysis and real-time fluorescence detection (Figure 6A,B).

Also, the weaker fluorescence response may indicate less efficient RNP complex formation or suboptimal amplification dynamics for the *stn* target. Nevertheless, the ability to generate a detectable, though modest, fluorescence signal demonstrates that the RPA–CRISPR/Cas12a system is still capable of identifying the *stn* gene, albeit with decreased sensitivity compared to the other virulence markers.

The combined data demonstrate that the RPA-CRISPR/Cas12a system possesses high analytical sensitivity, reliably detecting the target genes *pagN*, *siiD* and *sirA* at DNA concentrations as low as 10^2^ copies per reaction. The *stn* gene showed lower sensitivity, with detection limits of 10^4^ copies per reaction for plasmid DNA. Fluorescent signal intensity was highest for *pagN* and *siiD*, reflecting efficient RPA amplification and Cas12a activation. These results indicate that the system enables rapid, reproducible, and robust detection of *Salmonella enterica*, supporting its potential as a reliable tool for molecular diagnostics.

Taken together, these findings show that the RPA–CRISPR/Cas12a system reliably detects different virulence genes in separate reactions, demonstrating high sensitivity, rapid signal development, and robust performance across multiple *Salmonella* genetic targets.

### 3.4. Assessment of Assay Specificity

The analytical specificity of the method was experimentally evaluated using a panel of non-target bacteria. Genomic DNA of *Salmonella enterica* served as a positive control, while nuclease-free water was used as a negative control.

**Figure 7:**
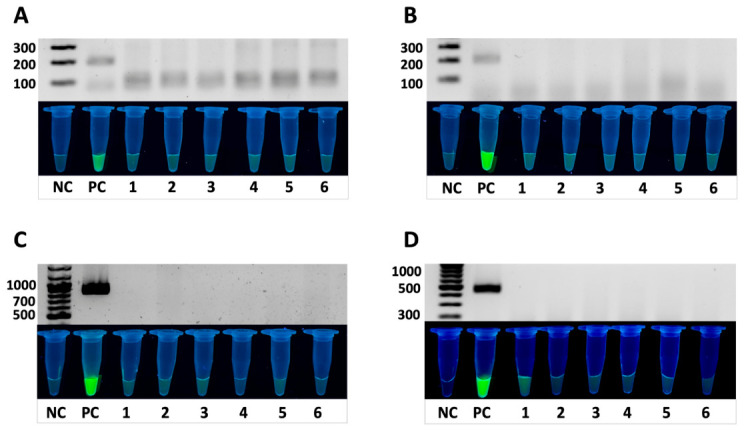
Analytical specificity assessment of the combined RPA-CRISPR/Cas12a system for detection of (**A**) *sirA*, (**B**) *stn*, (**C**) *siiD* and (**D**) *pagN* genes in *Salmonella enterica*. The upper panels show RPA amplification products analyzed by gel electrophoresis, while the lower panels display CRISPR/Cas12a-mediated FAM fluorescence. NC—negative control; PC—positive control. DNA from various bacterial strains was tested: 1—*A. baumannii*, 2—*P. aeruginosa*, 3—*E. coli*, 4—*S. aureus*, 5—*S. agalactiae*, 6—*K. pneumoniae*.

To determine the analytical specificity of the developed RPA-CRISPR/Cas12a system, a series of reactions was conducted using DNA from *Salmonella enterica* and six non-target bacterial species (Figure 7).

Following isothermal amplification (RPA), reaction products were analyzed by electrophoresis and subjected to visual detection using the Cas12a/crRNA complex targeting the *sirA*, *stn*, *siiD* and *pagN* genes. Detection was performed using a FAM-labeled fluorescent reporter.

A result was considered specific if a signal (gel band and fluorescence) was observed only in the positive control and absent in all non-target samples and the NTC. In the event of amplification in a non-target sample, testing was repeated in additional replicates; primer or crRNA redesign was performed if necessary.

As shown in Figure 7, intense green fluorescence was observed only in samples containing *S. enterica* DNA, indicating successful Cas12a activation and cleavage of the fluorescent reporter. No fluorescence was detected in samples containing DNA from other bacterial species, confirming the high specificity of the crRNAs and the absence of cross-reactivity. These results demonstrate that the combined RPA-CRISPR/Cas12a method provides accurate and specific detection of *Salmonella enterica* via the *sirA*, *stn*, *siiD* and *pagN* genes, making it a promising tool for rapid molecular diagnostics with high specificity.

Signal amplification was observed only in reactions containing *Salmonella enterica* DNA, while no amplification occurred with non-target species, demonstrating high analytical specificity of the developed RPA assay.

### 3.5. Validation of the Assay Using Bacterial Isolates

To assess the applicability of the developed RPA-CRISPR/Cas12a assay under conditions relevant to biological samples, genomic DNA from four *Salmonella enterica* isolates obtained from poultry sources was analyzed. Samples were prepared using a simplified thermal lysis protocol (95 °C for 5 min), and the resulting crude supernatant was used directly as a template for subsequent reactions.

The experimental workflow included an initial RPA amplification step (20 min), followed by a CRISPR/Cas12a trans-cleavage phase (10 min). Amplification of four target genes (*stn*, *siiD*, *sirA*, and *pagN*) from crude lysates was evaluated by agarose gel electrophoresis.

**Figure 8:**
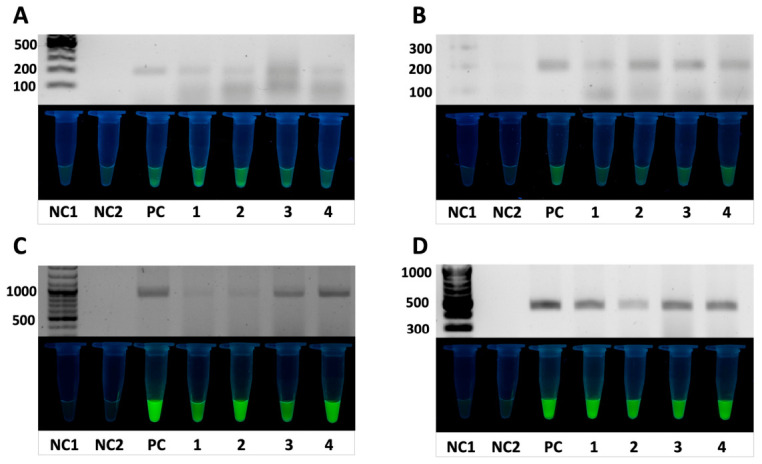
Validation of the RPA-CRISPR/Cas12a assay using bacterial isolates. Detection of target genes in *Salmonella enterica*: (**A**) *sirA*, (**B**) *stn*, (**C**) *siiD*, and (**D**) *pagN*. NC1—no-template negative control containing H_2_O; NC2—RPA negative control; PC—positive control, *S. enterica* ATCC 13311; samples 1, 2—*S. enterica* isolates; samples 3, 4—*Salmonella* spp. isolates.

As shown in Figure 8, bands corresponding to the expected amplicon sizes were observed for all targets across the tested isolates. Following amplification, the MbCas12a reaction mixture was added, and trans-cleavage activity was assessed using endpoint fluorescence detection, where fluorescence signals were observed in all isolate samples, while negative controls did not produce detectable signals. No evident non-specific amplification was detected under the experimental conditions.

**Figure 9:**
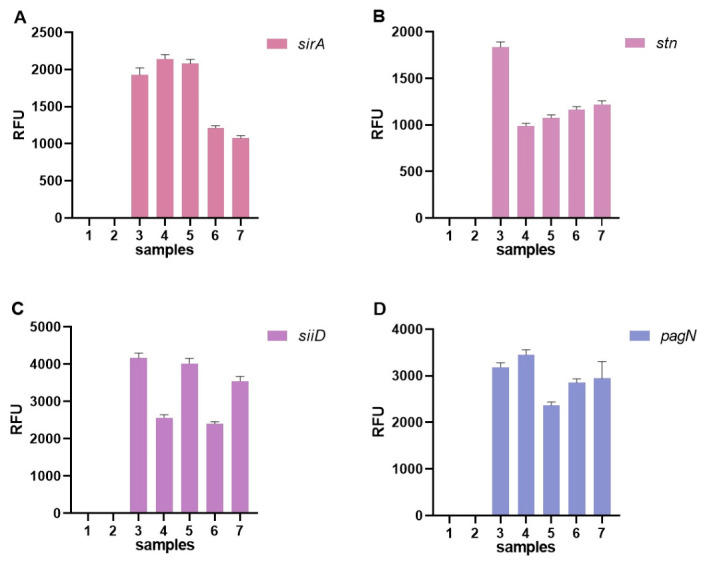
Fluorescence detection of *Salmonella* targets in control samples and bacterial isolates. (**A**) detection of *sirA* gene; (**B**) detection of *stn* gene; (**C**) detection of *siiD* gene; (**D**) detection of *pagN* gene. Samples: (1) negative control (H_2_O), (2) RPA negative control, (3) positive control (*S. enterica* ATCC 13311), (4–5) *S. enterica* isolates, and (6–7) *Salmonella* spp. isolates. Data are presented as mean ± SD (*n* = 3).

In addition, fluorescence signal dynamics were analyzed (Figure 9), demonstrating the development of detectable signals within the CRISPR/Cas12a reaction phase.

Fluorescence analysis demonstrated clear discrimination between negative controls (H_2_O and RPA), which showed only background signal, and the positive control (*S. enterica* ATCC 13311) as well as all tested isolates.

For all tested targets, strong fluorescence signals were observed in the positive control and in samples containing *Salmonella* isolates, whereas both negative controls showed background-level fluorescence.

Differences in signal intensity between targets were observed, with *siiD* and *pagN* generally producing higher RFU values compared to *sirA* and *stn*.

Data are presented as mean ± standard deviation (SD) based on three independent replicates (*n* = 3).

Taken together, these results indicate that the developed assay is compatible with DNA obtained via simplified extraction and is capable of detecting target sequences in *Salmonella* isolates under the tested conditions.

### 3.6. Portable Fluorescence Visualization Approach

**Figure 10:**
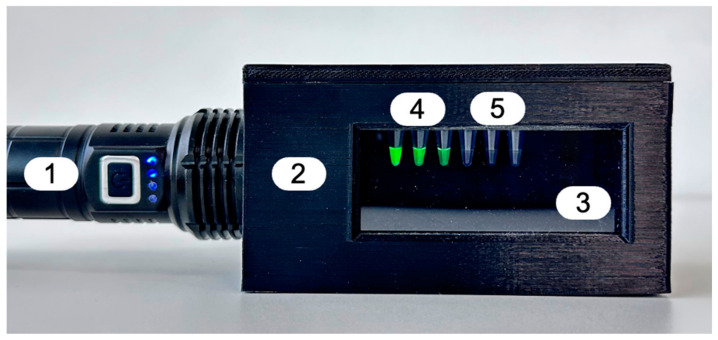
Portable fluorescence visualization approach for qualitative detection. The setup includes: (1) an ultraviolet light source; (2) a 3D-printed housing with integrated wells for sample placement; (3) a protective glass cover. Positive (4) and negative (5) control samples are shown.

The portable fluorescence visualization approach was implemented using a custom-designed 3D-printed housing fabricated from polymer material. The assembled setup and its key structural components are shown in Figure 10.

The housing was designed to accommodate reaction wells and to ensure stable positioning of samples during fluorescence visualization. An ultraviolet light source was integrated into the device to enable excitation of fluorescence signals generated during the CRISPR/Cas12a assay. All non-custom electronic components were commercially available and assembled without modification.

The visualization approach is intended as a simple and low-cost visualization tool for qualitative fluorescence detection. In practice, fluorescence can be visualized using a readily available ultraviolet light source, such as a standard handheld UV lamp. This enables a controlled dark environment and straightforward interpretation of results without the need for specialized instrumentation.

## 4. Discussion

**Figure 11:**
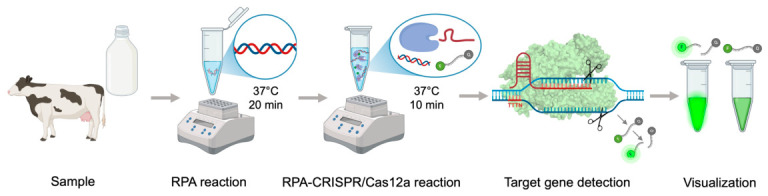
Workflow schematic of rapid target gene detection using the RPA-CRISPR/Cas12a system. Clinical material (e.g., milk) was first amplified by RPA at 37 °C for 20 min. The amplicons were then subjected to the CRISPR/Cas12a reaction at 37 °C for 10 min in the presence of the Cas12a–crRNA complex and a FAM-labeled ssDNA reporter. Target recognition activated Cas12a-mediated collateral cleavage of the reporter, generating a green fluorescence signal detectable under UV light or by lateral-flow assay.

The present study describes the development and validation of a rapid and highly sensitive RPA-CRISPR/Cas12a-based diagnostic platform for the detection of *Salmonella enterica.* The integration of isothermal amplification with CRISPR-mediated detection enables fast signal generation while maintaining high analytical specificity, supporting its potential application in decentralized and resource-limited settings (Figure 11). By significantly reducing detection time while maintaining or exceeding the sensitivity levels of traditional methods, this integrated approach holds the potential to transform existing food safety monitoring strategies and improve response efficiency during *Salmonella enterica* outbreaks. The potential impact of such innovations spans a wide range of domains—from strengthening public health and reducing economic losses to enhancing the efficiency of food production and processing operations.

A key strength of the proposed system lies in the use of multiple virulence-associated targets (*sirA*, *pagN*, *siiD*, and *stn*), which improves diagnostic robustness by reducing the risk of false-negative results associated with genetic variability among *Salmonella* strains. In this study, we developed an RPA–CRISPR/Cas12a system targeting four virulence-associated genes of *S. enterica*: *sirA* [40], *pagN* [41], *siiD* [42,43,44], and *stn* [45,46]. *SirA* encodes a transcriptional regulator controlling multiple virulence genes; *pagN* encodes an outer membrane protein essential for adhesion and invasion; *siiD* is a component of the type III secretion system encoded within *Salmonella* pathogenicity island 4 (SPI-4); and *stn* encodes *Salmonella* enterotoxin, a major factor in diarrheal disease. These genes were chosen because they play critical roles in pathogenesis and serve as reliable species-specific markers, allowing both accurate identification and virulence profiling of *S. enterica*. Compared to previously reported CRISPR/Cas12a-based detection systems for *Salmonella*, which typically rely on single genetic targets, the multiple target approach presented here provides an additional layer of reliability and may be particularly advantageous in heterogeneous or contaminated samples.

Analytical sensitivity testing demonstrated consistent detection of as few as 10^2^ DNA copies per reaction for *sirA*, *pagN*, and *siiD* using plasmid DNA templates. In contrast, *stn* showed lower and more variable sensitivity, with reliable detection achieved at 10^4^ copies per reaction. Differences in sensitivity and fluorescence signal intensity among the targets may be attributed to variations in amplification efficiency, target sequence characteristics, or template accessibility during the RPA and CRISPR/Cas12a reactions. The lower sensitivity observed for the *stn* target may also be influenced by differences in sequence context, including the efficiency of CRISPR/Cas12a activation. In particular, variations in PAM sequences and local structural features of the target region can affect enzyme recognition and trans-cleavage activity. In this study, targets such as *pagN* and *siiD*, which contain the PAM motif TTTA, consistently generated stronger fluorescence signals, suggesting more efficient Cas12a activation under the experimental conditions. These observations are consistent with our previous studies indicating that TTTA is an efficient PAM sequence for Cas12a systems derived from *Moraxella bovoculi*, facilitating effective target recognition and nuclease activity. However, the influence of PAM sequence and target structure on detection efficiency requires further investigation. Overall, these results highlight the importance of careful target and PAM selection when developing CRISPR/Cas12a-based diagnostic assays for *Salmonella enterica*.

While the initial sensitivity was established using idealized plasmid templates, the successful detection of all four genes in genomic DNA from poultry isolates demonstrates the assay’s translational potential. The use of a simple 5-min thermal lysis supernatant as a template highlights the robustness of the MbCas12a-based system against potential background matrix effects. This preliminary validation on confirmed *Salmonella* strains indicates that the platform can effectively bridge the gap between laboratory development and field-deployable applications, satisfying the basic requirements for rapid screening in agricultural settings.

The RPA-CRISPR/Cas12a system demonstrated excellent specificity, with no cross-reactivity observed for non-target bacterial species. The visual fluorescence readout, enabled by FAM-labeled reporters, allows rapid detection without sophisticated instrumentation. Furthermore, the use of a recombinant Cas12a protein derived from *Moraxella bovoculi* expands the diversity of CRISPR effectors used in diagnostics and demonstrates its applicability for sensitive nucleic acid detection.

Importantly, to support the development of simplified and potentially field-deployable applications, we additionally applied a portable fluorescence visualization approach featuring a 3D-printed housing with integrated sample wells and an ultraviolet light source for direct visual fluorescence detection. This provides a controlled environment for fluorescence visualization, ensuring stable sample positioning and enabling rapid differentiation between positive and negative samples under experimental conditions. This approach is intended as a proof-of-concept tool for qualitative signal detection. It’s simple and modular design may allow adaptation to different target genes or pathogens in future applications. However, further validation, including performance assessment under field conditions and comparison with standard detection systems, is required to fully evaluate its applicability for point-of-care use.

Overall, these findings indicate that the combination of rapid isothermal amplification, high CRISPR specificity, and portable fluorescence visualization offers a sensitive, reliable, and user-friendly method for the detection of *Salmonella enterica*, bridging laboratory diagnostics and field applications.

While the present study demonstrates the analytical feasibility of the proposed RPA-CRISPR/Cas12a platform, it does not yet constitute a fully validated diagnostic assay. Importantly, the primary objective of this work was to evaluate the applicability and performance of selected virulence gene targets (*sirA*, *pagN*, *siiD*, and *stn*) within a multi-target CRISPR-based detection framework, rather than to establish a fully standardized diagnostic system. Future work will aim to align the validation framework with established guidelines, including CLSI EP17-A2 for limit of detection determination, STARD reporting standards for diagnostic accuracy studies, and WHO target product profiles for molecular point-of-care tests. Such studies will include expanded strain panels, evaluation in complex sample matrices, and systematic assessment of diagnostic performance parameters.

## 5. Conclusions

The RPA-CRISPR/Cas12a system developed in this study enables the detection of *Salmonella enterica*. The platform was evaluated using four virulence markers: *pagN*, *siiD*, *sirA*, and *stn*. Analytical sensitivity experiments established detection limits for *pagN*, *siiD*, and *sirA* at 10^2^ copies per reaction in plasmid DNA templates, while the *stn* target was detected at 10^4^ copies. Fluorescent signal analysis showed that *pagN* and *siiD* produced higher signal intensities, whereas *sirA* and *stn* exhibited comparatively lower signals; however, all targets showed no cross-reactivity with non-target bacterial species under the tested conditions.

The applicability of the assay was evaluated using genomic DNA from four poultry-derived isolates (*S. enterica* and *Salmonella* spp.). Using a simplified thermal lysis (“boil-and-spin”) protocol (95 °C for 5 min), all four target genes were detected from crude supernatants. Consistent results were observed between agarose gel electrophoresis and visual fluorescence detection on a UV transilluminator, indicating that the MbCas12a-based detection is compatible with non-purified samples under the experimental conditions.

The integrated workflow allows detection within approximately 30 min (20 min for RPA and 10 min for CRISPR/Cas12a trans-cleavage) without the need for thermocycling. To support simplified analysis, a portable fluorescence visualization approach was applied as a proof-of-concept tool, featuring a 3D-printed housing and an integrated light source for visual differentiation between positive and negative samples. While this approach demonstrated consistent performance under laboratory conditions, the current version has several limitations. No direct benchmarking against standard fluorometric instruments was performed, and reproducibility under variable environmental conditions has not yet been systematically evaluated. In addition, the workflow timing analysis was limited to overall assay duration rather than device-specific performance metrics. Further validation using a larger number of isolates, diverse strain panels, and complex sample matrices is required to more fully assess the applicability of the proposed system for routine diagnostic use.

It should be noted that the portable fluorescence visualization approach is not intended as a dedicated analytical device, but rather as a simplified visualization approach. In practice, fluorescence detection can be performed using a readily available and low-cost ultraviolet light source, such as a standard handheld UV lamp. This design choice reflects the aim of enabling accessible and field-compatible detection without reliance on specialized instrumentation.

## Figures and Tables

**Table 1:** Oligonucleotides.

Method	Oligonucleotide	Sequence (5′ ⟶ 3′)
RPA primers	RPA_FW_Salmonella_siid	GTCAGGGCGTTATCACTACTAAAGATAATG
	RPA_RV_Salmonella_siid	TTCACATCGGCCAGCATAGTCCTTACTTTT
	RPA_FW_Salmonella_PagN	GCCTTTGTGTCTGCATCATAAGCGGTATGT
	RPA_RV_Salmonella_PagN	TTCCAGCTTCCAGTACGTTTAGAACTGGAT
	stn_fw_salmonella	GTATTCAGGCTGACCCGGACAGGCTGACTC
	stn_rv_salmonella	GCGTAAAAATCGCCTCCAGCTGATCCGGGG
	sirA_fw_salmonella	CTGAAATCTCATTGACCTTCTGACCCTTGG
	sirA_rv_salmonella	TCAGGAGGTGGTGAGCGCTATTCGTTCGGT
crRNA primers	crRNA_short	AATTCTAATACGACTCACTATAGGG
	crRNA_siid_SE_ttta	ATTCGCTTTTACCTCTTTATTGCTATCTACAAACAGTAGAAATTCCCTATAGTGAGTCGTATTAGAATT
	crRNA_sirA_SE_tttg	CATAATCTGCAACTCGCGTTCAGAATCTACAAACAGTAGAAATTCCCTATAGTGAGTCGTATTAGAATT
	crRNA_pagN_SE_ttta	TAATATTATGATTGACGCCAGTTAATCTACAAACAGTAGAAATTCCCTATAGTGAGTCGTATTAGAATT
	crRNA_stn_SE_tttc	TATCATCACTGTTACCGATAGCGGATCTACAAACAGTAGAAATTCCCTATAGTGAGTCGTATTAGAATT
	crRNA_siid_SE_tttt	TATATACGCCTGTTTCTTGTTAAGATCTACAAACAGTAGAAATTCCCTATAGTGAGTCGTATTAGAATT
	crRNA_sirA_SE_tttc	AGTCAGATTGAGCCTGCAAAAACGATCTACAAACAGTAGAAATTCCCTATAGTGAGTCGTATTAGAAT
	crRNA_sirA_SE_tttt	ATTGGCGGCCTTGAGGCGACGCGTATCTACAAACAGTAGAAATTCCCTATAGTGAGTCGTATTAGAATT
	crRNA_pagN_SE_tttc	ATTCCTGTTGGTTTTGGTATTAATATCTACAAACAGTAGAAATTCCCTATAGTGAGTCGTATTAGAATT
	crRNA_pagN_SE_tttg	TTGCCTGGGGCGCAGGTATCGGTGATCTACAAACAGTAGAAATTCCCTATAGTGAGTCGTATTAGAATT
	crRNA_pagN_SE_tttt	ATGCTGGCAAAGTAAGCATTTCAAATCTACAAACAGTAGAAATTCCCTATAGTGAGTCGTATTAGAATT
	crRNA_stn_SE_tttt	CCAGGGAACGATTAGCGTAGAGGCATCTACAAACAGTAGAAATTCCCTATAGTGAGTCGTATTAGAATT
Reporter	ssDNA reporter	FAM-TTATT-BHQ

## Data Availability

The data presented in this study are available from the corresponding author upon reasonable request.

## References

[1] Havelaar A.H., Kirk M.D., Torgerson P.R., Gibb H.J., Hald T., Lake R.J., Praet N., Bellinger D.C., de Silva N.R., Gargouri N. (2015). World Health Organization Global Estimates and Regional Comparisons of the Burden of Foodborne Disease in 2010. PLoS Med..

[2] Teklemariam A.D., Al-Hindi R.R., Albiheyri R.S., Alharbi M.G., Alghamdi M.A., Filimban A.A.R., Al Mutiri A.S., Al-Alyani A.M., Alseghayer M.S., Almaneea A.M. (2023). Human Salmonellosis: A Continuous Global Threat in the Farm-to-Fork Food Safety Continuum. Foods.

[3] Mkangara M. (2023). Prevention and Control of Human *Salmonella enterica* Infections: An Implication in Food Safety. Int. J. Food Sci..

[4] Lee S.Y., Oh S.W. (2024). Point-of-Care Diagnostic System for Viable *Salmonella* Species via Improved Propidium Monoazide and Recombinase Polymerase Amplification Based Nucleic Acid Lateral Flow. Diagnostics.

[5] Borovikov S., Ryskeldina A., Tursunov K., Syzdykova A., Akibekov O. (2023). Recombinant *Salmonella enterica* OmpX protein expression and its potential for serologically diagnosing *Salmonella* abortion in mares. Vet. World.

[6] Bakishev T., Amirgazin A., Kuibagarov M., Shevtsov A., Bakisheva Z., Yessembekova G., Kairzhanova A., Kadyrov A., Guo K., Wang X. (2025). Genome-wide characterization and comparative phylogenomics of three *Salmonella* Abortusequi strains isolated from equine abortions in Kazakhstan. Vet. World.

[7] Mahari S., Gandhi S. (2022). Recent Advances in Electrochemical Biosensors for the Detection of Salmonellosis: Current Prospective and Challenges. Biosensors.

[8] Patel A., Wolfram A., Desin T.S. (2024). Advancements in Detection Methods for *Salmonella* in Food: A Comprehensive Review. Pathogens.

[9] Feroci M., Grasso G., Dragone R., Curulli A. (2025). Electrochemical (Bio)Sensors for Toxins, Foodborne Pathogens, Pesticides, and Antibiotics Detection: Recent Advances and Challenges in Food Analysis. Biosensors.

[10] Chakraborty J., Chaudhary A.A., Khan S.U., Rudayni H.A., Rahaman S.M., Sarkar H. (2022). CRISPR/Cas-Based Biosensor As a New Age Detection Method for Pathogenic Bacteria. ACS Omega.

[11] Chen J.S., Ma E., Harrington L.B., Da Costa M., Tian X., Palefsky J.M., Doudna J.A. (2018). CRISPR-Cas12a target binding unleashes indiscriminate single-stranded DNase activity. Science.

[12] Gootenberg J.S., Abudayyeh O.O., Lee J.W., Essletzbichler P., Dy A.J., Joung J., Verdine V., Donghia N., Daringer N.M., Freije C.A. (2017). Nucleic acid detection with CRISPR-Cas13a/C2c2. Science.

[13] Zhuang L., Gong J., Zhang P., Zhang D., Zhao Y., Yang J., Liu G., Zhang Y., Shen Q. (2024). Research progress of loop-mediated isothermal amplification in the detection of *Salmonella* for food safety applications. Discov. Nano.

[14] Zhao Y., Chen F., Li Q., Wang L., Fan C. (2015). Isothermal Amplification of Nucleic Acids. Chem. Rev..

[15] Mei X., Zhai X., Lei C., Ye X., Kang Z., Wu X., Xiang R., Wang Y., Wang H. (2019). Development and application of a visual loop-mediated isothermal amplification combined with lateral flow dipstick (LAMP-LFD) method for rapid detection of *Salmonella* strains in food samples. Food Control.

[16] Liu L., Zhao G., Li X., Xu Z., Lei H., Shen X. (2022). Development of rapid and easy detection of *Salmonella* in food matrics using RPA-CRISPR/Cas12a method. LWT.

[17] Zendrini A., Carta V., Filipello V., Ragni L., Cosciani-Cunico E., Arnaboldi S., Bertasi B., Franceschi N., Ajmone-Marsan P., De Medici D. (2021). One-Day Molecular Detection of *Salmonella* and *Campylobacter* in Chicken Meat: A Pilot Study. Foods.

[18] Aman R., Mahas A., Mahfouz M. (2020). Nucleic Acid Detection Using CRISPR/Cas Biosensing Technologies. ACS Synth. Biol..

[19] Mao Z., Chen R., Wang X., Zhou Z., Peng Y., Li S., Han D., Li S., Wang Y., Han T. (2022). CRISPR/Cas12a-based technology: A powerful tool for biosensing in food safety. Trends Food Sci. Technol..

[20] Selvam K., Ahmad Najib M., Khalid M.F., Ozsoz M., Aziah I. (2022). CRISPR-Cas Systems-Based Bacterial Detection: A Scoping Review. Diagnostics.

[21] Li S.Y., Cheng Q.X., Wang J.M., Li X.Y., Zhang Z.L., Gao S., Cao R.B., Zhao G.P., Wang J. (2018). CRISPR-Cas12a-assisted nucleic acid detection. Cell Discov..

[22] Mao X., Zhao Y., Jiang J., Du Q., Tu B., Li J., Wang F. (2022). Sensitive and high-accuracy detection of *Salmonella* based on CRISPR/Cas12a combined with recombinase polymerase amplification. Lett. Appl. Microbiol..

[23] Yuan J., Wang L., Huang L., He K., Wang H., Xu X., Su B., Wang J. (2024). CRISPR-Cas12a-Mediated Hue-Recognition Lateral Flow Assay for Point-of-Need Detection of *Salmonella*. Anal. Chem..

[24] Jeong Y., Lee J., Choi S., Shin D., Jang S., Son S.U., Kang T., Jung J., Hwang J., Lim E.K. (2026). On-site detection of airborne foodborne pathogens using a field-deployable recombinase polymerase amplification and CRISPR/Cas12a cleavage activity assay. Biosens. Bioelectron..

[25] Kachwala M.J., Hamdard F., Cicek D., Dagci H., Smith C.W., Kalla N., Yigit M.V. (2024). Universal CRISPR-Cas12a and Toehold RNA Cascade Reaction on Paper Substrate for Visual *Salmonella* Genome Detection. Adv. Healthc. Mater..

[26] Wei Z., Zhang L., Wang Y., Xu X., Cao L., Lin H., Sui J., Wang K., Wang X. (2025). Development of a Label-Free Colorimetric and Fluorescent Diagnostic Platform for Foodborne *Salmonella* Based on RPA-CRISPR/Cas12 Assay in a Single Tube. J. Agric. Chem..

[27] Han D.H., Lee S.Y., Kim Y., Oh J., Park J., Park Y.M., Kim S.G., Kim T.S., Park J.K. (2026). Ultrasensitive Detection of Multiple Foodborne Pathogens Using CRISPR-Cas12a on a Finger-Actuated Microfluidic Device Integrated with a Modular Pressurizing Pump. Anal. Chem..

[28] Sui Z., Chen B., Zhao J., Deng R., Xu J. (2026). Pronounced Fluorescence Polarization Enhancement Driven by RPA-CRISPR/Cas12a Induced Nucleoprotein Assembly for *Salmonella* Analysis in Animal-Derived Food Matrices. Anal. Chem..

[29] Zhuang J., Zhao Z., Lian K., Yin L., Wang J., Man S., Liu G., Ma L. (2022). SERS-based CRISPR/Cas assay on microfluidic paper analytical devices for supersensitive detection of pathogenic bacteria in foods. Biosens. Bioelectron..

[30] Huang Y., Liang W., Huang M., Deng Y., Huang Z., Ai C., Tan W., Jiang L. (2026). Application of CRISPR/Cas13a system on the rapid detection of *Salmonella* spp. PLoS Neglected Trop. Dis..

[31] Wang Y., Du P., Shao Y., Wang W., Liu Y., Ma Y., Hu P., Cao J., Wang X., Abd El-Aty A.M. (2024). An Innovative and Efficient Fluorescent Detection Technique for *Salmonella* in Animal-Derived Foods Using the CRISPR/Cas12a-HCR System Combined with PCR/RAA. J. Agric. Chem..

[32] Li L., Xiong Y., Guo Y., Duan H., Leng Y., Huang X., Chen G., Xiong Y. (2025). G-Quadruplex-Enhanced DNA Silver Nanoclusters Enable CRISPR/Cas12a System for Ultrasensitive Detection of *Salmonella* typhimurium. J. Agric. Chem..

[33] Guo Y., Guo W., Wu Z., Xu H., Zhang X., Zou X., Sun Z. (2025). A Microfluidic Chip-Based Electrochemical Biosensor Coupled with CRISPR/Cas12a for Simultaneous Detection of Foodborne Pathogens. Anal. Chem..

[34] Chen Q., Wang H., Xu H., Peng Y., Yao B., Chen Z., Yang J., Adeloju S., Chen W. (2025). One-pot RPA-CRISPR/Cas12a integrated dual-mode electrochemical lateral flow strip for ultrasensitive and precise detection of *Salmonella*. Biosens. Bioelectron..

[35] Liu Y., Chao Z., Ding W., Fang T., Gu X., Xue M., Wang W., Han R., Sun W. (2024). A multiplex RPA-CRISPR/Cas12a-based POCT technique and its application in human papillomavirus (HPV) typing assay. Cell. Mol. Biol. Lett..

[36] Gu X., Tang Q., Kang X., Ji H., Shi X., Shi L., Pan A., Zhu Y., Jiang W., Zhang J. (2024). A portable CRISPR-Cas12a triggered photothermal biosensor for sensitive and visual detection of *Staphylococcus aureus* and *Listeria monocytogenes*. Talanta.

[37] Shaizadinova A., Amanzholova M., Kirillov S., Bulashev A., Abeldenov S. (2023). Rapid and highly sensitive LAMP-CRISPR/Cas12a-based identification of bovine mastitis milk samples contaminated by *Escherichia coli*. J. Agric. Food Res..

[38] Amanzholova M., Shaizadinova A., Bulashev A., Abeldenov S. (2024). Genetic identification of *Staphylococcus aureus* isolates from cultured milk samples of bovine mastitis using isothermal amplification with CRISPR/Cas12a-based molecular assay. Vet. Res. Commun..

[39] Shaizadinova A., Amanzholova M., Rukavitsina I., Abeldenov S., Zhumakayev A.R. (2024). CRISPR/Cas12a-based method coupled with isothermal amplification to identify *Alternaria* spp. isolated from wheat grain samples. Front. Microbiol..

[40] Ahmer B.M., van Reeuwijk J., Watson P.R., Wallis T.S., Heffron F. (1999). *Salmonella* SirA is a global regulator of genes mediating enteropathogenesis. Mol. Microbiol..

[41] Mlangeni L.N., Ramatla T., Lekota K.E., Price C., Thekisoe O., Weldon C. (2024). Occurrence, Antimicrobial Resistance, and Virulence Profiles of *Salmonella* Serovars Isolated from Wild Reptiles in South Africa. Int. J. Microbiol..

[42] Xu C., Ren X., Feng Z., Fu Y., Hong Y., Shen Z., Zhang L., Liao M., Xu X., Zhang J. (2017). Phenotypic Characteristics and Genetic Diversity of *Salmonella enterica* Serotype Derby Isolated from Human Patients and Foods of Animal Origin. Foodborne Pathog. Dis..

[43] Osman K.M., Hassan W.M., Mohamed R.A. (2014). The consequences of a sudden demographic change on the seroprevalence pattern, virulence genes, identification and characterisation of integron-mediated antibiotic resistance in the *Salmonella enterica* isolated from clinically diarrhoeic humans in Egypt. Eur. J. Clin. Microbiol. Infect. Dis. Off. Publ. Eur. Soc. Clin. Microbiol..

[44] Petano-Duque J.M., Rueda-García V., Rondón-Barragán I.S. (2023). Virulence genes identification in *Salmonella enterica* isolates from humans, crocodiles, and poultry farms from two regions in Colombia. Vet. World.

[45] Mahnaz J.S., Soheila M.B., Pejvak K. (2023). Assessment of stn, sipB and sopB Virulence Genes in Various *Salmonella* Serovars. Arch. Razi Inst..

[46] Moore M.M., Feist M.D. (2007). Real-time PCR method for *Salmonella* spp. targeting the stn gene. J. Appl. Microbiol..

